# Flecainide‐Induced Interstitial Lung Disease: A Case Report and Review of the Literature

**DOI:** 10.1002/ccr3.72486

**Published:** 2026-04-24

**Authors:** Elizabeth Jenkins, John McGinty, Brian Skinner

**Affiliations:** ^1^ Reid Health Richmond Indiana USA; ^2^ Marian University Tom & Julie Wood College of Osteopathic Medicine Indianapolis Indiana USA

**Keywords:** adverse drug events, antiarrhythmic, flecainide, interstitial lung disease, pulmonary toxicity

## Abstract

This case describes a rare adverse effect in which the subject developed interstitial lung disease after initiating a class IC antiarrhythmic, flecainide. There is no current treatment recommendation for flecainide‐induced interstitial lung disease. Previously reported cases were successful with discontinuation of the offending drug and initiation of systemic corticosteroids; however, this patient did not respond to this treatment plan, resulting in death. Obtaining a thorough patient history regarding new medications and the start of symptoms played a crucial role when creating a differential diagnosis for this case.

## Key Clinical Message

Although rare, flecainide may cause drug‐induced interstitial lung disease which can have fatal complications. Diagnosis requires exclusion of other potential causes quickly as prompt drug discontinuation of flecainide and initiation of steroids are key components of treatment.

## 1 Introduction

Interstitial lung disease is a broad term used to define a large group of chronic lung disorders. General pulmonary pathology features include inflammation and progressive, irreversible scarring around the alveoli which inhibits the ability to exchange oxygen in the lungs. Drug‐induced interstitial lung disease (DIILD) is a subtype that falls into this large array of diseases. DIILD occurs due to a hypersensitivity reaction when the lungs become inflamed and fibrotic after exposure to a specific drug [1]. While not completely understood, it is thought that the underlying pathophysiology is a combination of direct cell injury to pneumonocytes and alveolar epithelial cells in addition to an immune‐mediated response leading to the fibrosing of lung tissue. Anti‐arrhythmic drugs such as procainamide, tocainide, amiodarone, and flecainide have been identified as risk factors for developing DIILD, although the mechanism is unclear [2].

## 2 Methods

### 2.1 Informed Consent and Ethics Approval

Written informed consent was obtained from the patient's next of kin, as the patient was deceased, in accordance with the journal's patient consent policy. Additionally, approval for publication was sought and received from the hospital's Institutional Review Board.

### 2.2 Search Strategy and Selection Criteria

A comprehensive search of publications was conducted through July 2025 in three databases: PubMed, Google Scholar, and Scopus. The primary search method was (((“Interstitial Lung Disease”) OR (DIILD) OR (“Drug Induced Pulmonary Toxicity”) OR (“pneumonitis”)) AND (flecainide)). Relevant publications were manually searched to identify any potential additional publications. All published case reports, case series, articles, or conference abstracts that include patient‐specific information relating to flecainide and any incidence of pulmonary toxicity were included, and publications without English availability were excluded. Two authors independently reviewed all articles and performed a comparative analysis to determine appropriate inclusion and exclusion.

## 3 Results

### 3.1 Case Presentation

A 78‐year‐old male presented to the emergency department (ED) with a chief complaint of worsening fatigue and weakness after a recent hospitalization for pneumonia. Past medical history included hypertension, atrial fibrillation, gastroesophageal reflux disease, benign prostatic hypertrophy, anxiety, obstructive sleep apnea, and hyperlipidemia. The patient was a former smoker but had not used tobacco products in over 45 years. Medications at time of admission included flecainide, atenolol, apixaban, lisinopril, and amlodipine. He was previously hospitalized and discharged 5 days prior due to sepsis secondary to viral pneumonia. The patient had a productive cough with brown sputum and occasionally quarter‐sized bright red mucus. He became hypoxic in the ED with an oxygen saturation of 85% on 4 L of oxygen. Of note, flecainide had been initiated 6 weeks before this admission with respiratory symptoms appearing approximately 2–3 weeks after drug initiation. Flecainide was initiated at the lowest starting dose (50 mg twice daily), which was appropriate for both renal and hepatic function. The patient had been on stable doses of his other chronic medications before his admission, and no other medications had been recently initiated or discontinued before symptom onset. An abdominal computed tomography (CT) scan was incidentally taken around that time and revealed no evidence of chronic pulmonary disease in the bases of the lungs.

### 3.2 Diagnostic Investigation

Initial laboratory work was remarkable for a white blood cell count of 14.7 × 109 cells/L, sodium 125 mEq/L, and a troponin value of 0.05 ng/mL. A chest CT with angiography revealed extensive bilateral infiltrates with small bilateral effusions without focal mass, cavitation, or nodular lesions to suggest malignancy or focal infectious processes. Chest CT without contrast a week before admission revealed pulmonary infiltrates along with old granulomatous disease with underlying chronic lung disease that was not present in studies from a few months prior.

Given the unclear etiology for the patient's acute hypoxic respiratory failure, a multitude of diagnoses were considered including infectious etiologies, autoimmune (i.e., vasculitis), DIILD, malignancy, and cardiovascular causes. 1,3‐Beta‐D‐Glucan, blood cultures, legionella antigen, pneumococcal antigen, and viral respiratory panel were all negative. Additionally, ANA and ANCA were also negative. An echocardiogram revealed a normal ejection fraction with grade 2 diastolic dysfunction. A detailed history was obtained, and it was noted that the patient began to complain of shortness of breath and cough in the weeks following flecainide initiation. A transbronchial biopsy was obtained and was initially read as indicating a fibrotic lung with emphysematous change, numerous fibroblastic foci, and calcified/hyalinized granulomata with no evidence of oncologic disease. The pathology results were submitted to a university hospital for a second opinion, and an overread additionally noted diffuse alveolar damage without histopathologic evidence of infection, vasculitis, or malignancy. The patient was diagnosed with DIILD with notable diffuse alveolar damage. The likelihood of a drug‐related adverse reaction was evaluated using the Naranjo Adverse Drug Reaction Probability Scale [3]. The Naranjo scale is widely utilized to assess the likelihood that a drug caused an adverse event by assigning weighted scores to various criteria. Based on the total score, the relationship can be categorized as definite, probable, possible, or doubtful. In this case, the Naranjo score was calculated to be 6, indicating a probable relationship between flecainide and the lung injury in this patient.

### 3.3 Literature Review

**FIGURE 1:**
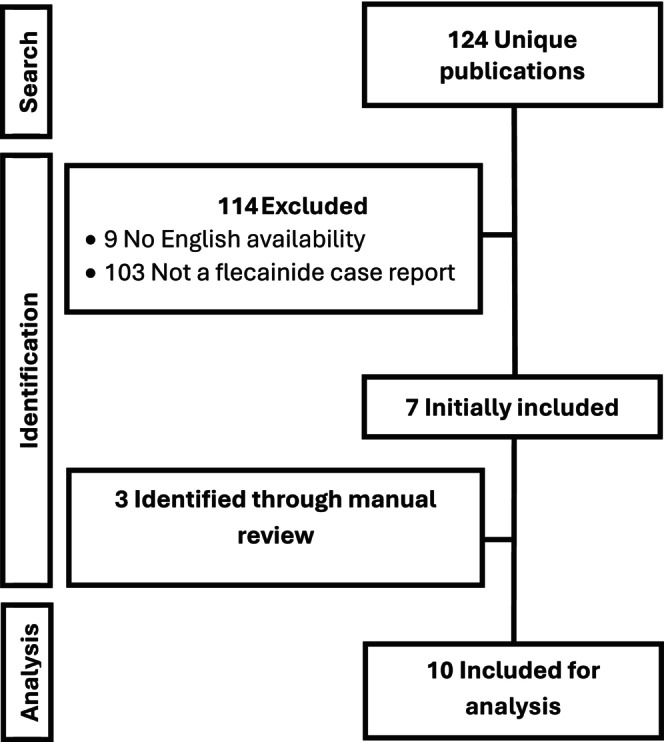
Flow‐diagram of study selection.

The initial search yielded 124 unique publications to be screened for potential inclusion, with 7 published reports identified. The majority of excluded publications were review articles or case reports on other medications (90.3%). A manual review of citations in relevant literature and the published reports identified yielded an additional 3 publications for inclusion for a total of 10 publications with 12 cases in total, including this case (see Figure 1).

Of the 12 available case reports, mortality was relatively high with 4 of the 12 patients (33.3%) dying despite flecainide withdrawal and treatment with corticosteroid therapy (see Table 1) [4, 5, 6, 7, 8, 9, 10, 11, 12, 13]. Among the surviving cohort, both clinical and radiological improvement were consistently seen within 2 weeks to 3 months after stopping flecainide therapy. Flecainide was withdrawn in all cases, and corticosteroids were used in 11 of the 12 cases (91.7%), with a commonly reported regimen of prednisone or methylprednisolone at 1 mg/kg/day, although variation was noted as some used fixed‐dosing regimens and others did not specify the specific regimen administered. In general, corticosteroid usage resulted in a potentially faster recovery as the one steroid‐free case saw a more gradual improvement at 2.5 months [4].

**TABLE 1:** Summary of published case reports.

Primary Author (Year)	Patient Demographics	Dose	Exposure Duration	Pulmonary Complication	Flecainide discontinued	Steroids	Outcome
Akoun (1991) [4]	61 M	200 mg daily	15 months	Interstitial pneumonitis	Yes	None	Clinical, radiological, and PFT improvement at 2.5 months
Hanston (1991) [5]	49 M	150 mg twice daily	12 months	Interstitial pneumonitis	Yes	Methylprednisolone 1 mg/kg daily	Death (day 66)
Robain (2000) [6]	59 M	NR	NR; 60 g cumulative exposure	Interstitial pneumonitis	Yes	Prednisone 1 mg/kg	Clinical and radiological improvement at day 15
Pesenti (2002) [7]—Case 1	75 M	100 mg daily	22 months	NSIP	Yes	Prednisone	Clinical and radiological improvement at 1 month
Pesenti (2002) [7]—Case 2	73 M	100 mg daily	4 months	NSIP	Yes	Prednisone	Clinical improvement after 2 weeks; Radiological improvement at 2 months
Huang (2018) [8]	70F	100 mg twice daily	1 day	Interstitial pneumonitis	Yes	Methylprednisolone 1 mg/kg/day	Clinical improvement after 2 months; Radiological improvement at 3 months
Najjar (2019) [9]	59F	NR	NR	Diffuse alveolar hemorrhage	Yes	Methylprednisolone	Clinical, radiological, and PFT improvement (timeframe NR)
Godil (2020) [10]	80 M	50 mg twice daily	4 years	Drug‐induced organizing pneumonia	Yes	Prednisone 50 mg daily	Clinical and radiological improvement at week 3
Moureau (2022) [11]	61F	100 mg daily	28 months	Drug‐induced organizing pneumonia	Yes	Methylprednisolone 1.25 mg/kg x 11 days then 1 mg/kg	Death (day 21)
Johri (2023) [12]	65F	NR	7 months	Diffuse alveolar damage	Yes	Methylprednisolone 1 mg/kg/day	Death (3 weeks)
Ferrer‐Pargada (2024) [13]	74F	100 mg daily	2 months	Interstitial pneumonitis	Yes	Prednisone 30 mg	Clinical, radiological, and PFT improvement at week 4
This case	78 M	50 mg twice daily	6 weeks	Interstitial pneumonitis with diffuse alveolar damage	Yes	Methylprednisolone 40 mg every 12 h then dexamethasone 10 mg every 8 h	Death (day 13)

Abbreviations: NR = not reported, PFT = pulmonary function testing, NSIP = non‐specific interstitial pneumonia.

Latency between exposure and symptom onset varied dramatically with the shortest interval occurring just 1 day after initiation whereas the longest exposure was approximately 4 years [8, 10]. Most cases that reported the duration of exposure before symptom onset, however, occurred between 2 to 22 months (7/10, 70%).

The pattern of pulmonary injury, based on radiological and/or histological findings, was heterogeneous with some clustering. Interstitial pneumonitis was most frequent (6/12, 50%) followed by non‐specific interstitial pneumonia and drug‐induced organizing pneumonia with 2 case reports each (16.7%). Alveolar damage and/or hemorrhage was specifically noted in 25% of the total cases and comprised 66.7% of fatal cases.

### 3.4 Intervention and Outcome

Broad spectrum antibiotics were initiated empirically in the ED. The patient's respiratory status continued to deteriorate with oxygen requirements increasing to 12 L. Upon recognition of DIILD, flecainide was discontinued, and the patient was initiated on methylprednisolone 40 mg intravenously every 12 h and subsequently transitioned to dexamethasone 10 mg intravenously every 8 h. Of note, this dosing regimen is lower than the usual 1 mg/kg/day of methylprednisolone or prednisone reported in other cases. As the respiratory status continued to worsen, the patient was then placed on BiPAP and ultimately intubated.

During a breathing trial, the patient developed supraventricular tachycardia leading to cardiogenic shock. The patient underwent emergent cardioversion. Thereafter, he was transitioned to comfort care the next day and extubated. The patient expired approximately 3 h following extubation.

## 4 Discussion

Although few case reports of flecainide‐associated DIILD have been published, fatal outcomes have been noted in two previous cases [11, 12]. Treatment of DIILD generally involves discontinuation of the offending agent and initiation of systemic corticosteroids. Many of the signs and symptoms from our case align with the few presentations that have been published on flecainide‐induced interstitial lung disease. It has previously been characterized as an idiosyncratic cell‐mediated immunologic reaction. This proposed mechanism is supported by a prior case report describing two patients in which bronchoalveolar lavage demonstrated lymphocytosis, eosinophilia, and an inverted CD4/CD8 ratio. The presence of an inverted CD4/CD8 ratio further supports an immune‐mediated pathogenesis, rather than direct insult to the alveolar epithelium [1, 7]. Alternatively, flecainide has been shown to persist in lung tissue up to 66 days following withdrawal. Additionally, autopsy of another patient found markedly elevated levels of flecainide in comparison to other tissues. Taken together, the risk for direct cytotoxic injury cannot be excluded [11]. While this patient presented within a few months of flecainide initiation, some case reports have found that symptoms may develop acutely within hours of administration, particularly if a patient has received other drugs known to cause pulmonary toxicity [8]. It should be noted that over 380 medications may cause drug‐induced respiratory diseases. While the time course of this presentation suggests it most likely may have been caused by flecainide, investigators are unable to rule out other potential drug‐induced causes, such as lisinopril [2].

## 5 Conclusion

This case adds to the growing body of literature suggesting flecainide may cause DIILD, as only limited case reports have been published regarding the risk of flecainide‐induced pulmonary toxicity. Clinicians should be reminded that a detailed history and timeline are crucial to the work‐up of DIILD, although a definitive diagnosis still requires exclusion of other potential causes. Upon recognition, prompt discontinuation of flecainide and initiation of systemic corticosteroids are the mainstays of therapy.

## Author Contributions

**Elizabeth Jenkins:** conceptualization, data curation, writing – original draft, writing – review and editing. **John McGinty:** investigation, methodology, supervision, writing – review and editing. **Brian Skinner:** conceptualization, formal analysis, funding acquisition, software, supervision, visualization, writing – original draft, writing – review and editing.

## Funding

Support for publication costs were provided by Marian University.

## Ethics Statement

Ethics approval was obtained through Reid Health's IRB. IORG 001967, IRB# 00002478, Federalwide Assurance Number: FWA00002676.

## Consent

Written consent for publication was obtained from the patient's next of kin, as the patient was deceased.

## Conflicts of Interest

The authors declare no conflicts of interest.

## Data Availability

The data that supports the findings within this article are openly available in Table 1.
